# O.2.3-4 A standardised core outcome set for measurement and reporting sedentary behaviour interventional research: the CROSBI consensus study

**DOI:** 10.1093/eurpub/ckad133.130

**Published:** 2023-09-11

**Authors:** Fiona M Curran, Kieran P Dowd, Casey L Peiris, Hidde P van der Ploeg, Mark S Tremblay, Grainne O’Donoghue

**Affiliations:** School of Public Health, Physiotherapy and Sports Science, University College Dublin, D04 V1W8 Dublin, Ireland; Department of Sport and Health Sciences, Technological University of Shannon, V94 EC5T Athlone, Irelan; Department of Physiotherapy, School of Allied Health, Human Services and Sport, La Trobe University, Melbourne 3086, Australia; Amsterdam UMC, Department of Public and Occupational Health, Amsterdam Public Health Research Institute, Vrije Universiteit Amsterdam, van der Boechorststraat 7, 1081 BT Amsterdam, The Netherlands; hp.vanderploeg@amsterdamumc.nl; Children’s Hospital of Eastern Ontario Research Institute, Ottawa, ON K1H 8L1, Canada; Department of Pediatrics, University of Ottawa, ON K1N 6N5, Canada; Department of Health Sciences, Carleton University, Ottawa, ON K1S 5B6, Canada; fiona.curran@ucd.ie; School of Public Health, Physiotherapy and Sports Science, University College Dublin, D04 V1W8 Dublin, Ireland

## Abstract

**Purpose:**

Heterogeneity of descriptors and outcomes measured and reported in sedentary behaviour (SB) research hinder the meta-analysis of data and accumulation of evidence. The objective of the Core Research Outcomes for Sedentary Behaviour Interventions (CROSBI) consensus study was to identify and validate, a core outcome set (COS) to report (what, how, when to measure) in interventional sedentary behaviour studies.

**Methods:**

Outcomes, extracted from a systematic literature review, were categorized into domains and data items (COS v0.0). International experts (n = 5) provided feedback and identified additional items, which were incorporated into COS v0.1. A two round online Delphi survey was conducted to seek consensus from a wider stakeholder group and outcomes that achieved consensus in the second round COS (v0.2), were ratified by the expert panel.

**Results:**

The final COS (v1.0) contains 53 data items across 12 domains, relating to demographics, device details, wear-time criteria, wear-time measures, posture-related measures, sedentary breaks, sedentary bouts and physical activity. Notably, results indicate that sedentary behaviour outcomes should be measured by devices that include an inclinometry or postural function.

**Conclusions:**

The proposed standardised COS is available openly to enhance the accumulation of pooled evidence in future sedentary behaviour intervention research and practice.

